# Critical roles of time-scales in soft tissue growth and remodeling

**DOI:** 10.1063/1.5017842

**Published:** 2018-06-05

**Authors:** Marcos Latorre, Jay D. Humphrey

**Affiliations:** 1 Escuela Técnica Superior de Ingeniería Aeronáutica y del Espacio, Universidad Politécnica de Madrid, 28040 Madrid, Spain; 2 Department of Biomedical Engineering, Yale University, New Haven, Connecticut 06520, USA; 3 Vascular Biology and Therapeutics Program, Yale School of Medicine, New Haven, Connecticut 06520, USA

## Abstract

Most soft biological tissues exhibit a remarkable ability to adapt to sustained changes in mechanical loads. These macroscale adaptations, resulting from mechanobiological cellular responses, are important determinants of physiological behaviors and thus clinical outcomes. Given the complexity of such adaptations, computational models can significantly increase our understanding of how contributions of different cell types or matrix constituents, and their rates of turnover and evolving properties, ultimately change the geometry and biomechanical behavior at the tissue level. In this paper, we examine relative roles of the rates of tissue responses and external loading and present a new rate-independent approach for modeling the evolution of soft tissue growth and remodeling. For illustrative purposes, we also present numerical results for arterial adaptations. In particular, we show that, for problems defined by particular characteristic times, this approximate theory captures well the predictions of a fully general constrained mixture theory at a fraction of the computational cost.

## I. INTRODUCTION

As aptly stated in 1995 by Fung, “Every specialty in biomechanics begins with the study of constitutive relations,”^1^ that is, descriptors of material behavior for particular conditions of interest. Among his many contributions, Fung showed that although soft tissues tend to exhibit nonlinearly viscoelastic behaviors under certain conditions, they can often be regarded as pseudoelastic under physiological conditions. Indeed, it is for this reason that preconditioning is a fundamental part of most experimental protocols for studying the biomechanical behavior of soft tissues and the vast majority of constitutive relations for stress are based on hyperelasticity, not viscoelasticity. When considering the remarkable ability of soft tissues to respond to changing mechanical conditions, that is to grow and remodel, Fung stated further that “the scope of constitutive equations broadens: it should now include a mass-and-structure growth-stress relationship as well as a stress-strain relationship.” For the past 20+ years, a key specialty area of investigation in soft tissue mechanics has focused on developing and testing constitutive relations for growth and remodeling (G&R).

Of the different approaches available, we have advocated and employed a constrained mixture theory for soft tissue G&R.^2^ Briefly, this approach requires identification of three classes of constitutive relations: a hyperelastic descriptor of the mechanical behavior of each of the structurally significant constituents, as well as descriptors for mass density production rates and related survival functions for these same constituents. The mechanical properties and rates of production and removal of the individual constituents can depend, in part, on the state of stress/strain at which they were incorporated within the extant tissue, as well as on the current state of stress/strain. Hence, this approach is consistent with Fung's call for “growth-stress” relations. A special case of tissue maintenance exists when rates of production and removal balance perfectly while constituent's turnover in an unchanging mechanical state. Finally, the term “constrained” implies that all motions of each constituent correspond with those of the mixture even though each constituent can possess an individual natural (i.e., stress-free) configuration. This constrained mixture approach has proven useful in describing a host of evolving vascular conditions, including the development and resolution of cerebral vasospasms, mechano-adaptation of arteries to altered blood pressure and flow, arterial aging, the enlargement of aneurysms, the development of tissue engineered vascular grafts, and the maladaptation of vein grafts,^3–8^ as well as other cellular and tissue-level processes.^9–11^

Because every cell and structurally significant constituent has a finite half-life and presumed memory associating its loss to the state of stress at which time the constituent was incorporated within the extant matrix, the classical constrained mixture theory uses a hereditary integral formulation similar to that of nonlinear viscoelasticity.^2^ Albeit motivated directly by the mechanobiology, this integral formulation can be expensive computationally, including the need to store all past states over which constituents were deposited. For this reason, there has been a search for suitable simplifications that preserve advantages of the mixture approach (e.g., the ability to account for material properties and rates of turnover inherent to the different constituents that constitute the tissue) while improving computational efficiency. One approach has been to introduce a temporal homogenization^12^ while another has been to derive an associated steady-state form that reveals the final (evolved) state,^13^ not unlike equivalence derivations in viscoelasticity that relate integral and rate forms^14^ or those that focus on long-term responses in relaxation and creep.^15^ In contrast, in this paper we consider time scales inherent to the rates of mechanical loading and G&R responses to determine conditions under which a rate-independent (“pseudoelastic”) theory can hold throughout G&R. In this sense, our current formulation is similar to concepts introduced by Fung to model tissues that exhibit viscoelastic behaviors using concepts of hyperelasticity. For purposes of illustration and application, we use this theory to simulate arterial responses to altered pressure, flow, and axial stretch.

## II. RESULTS

### A. Long-term, steady-state, tissue maintenance solution

**FIG. 1.**
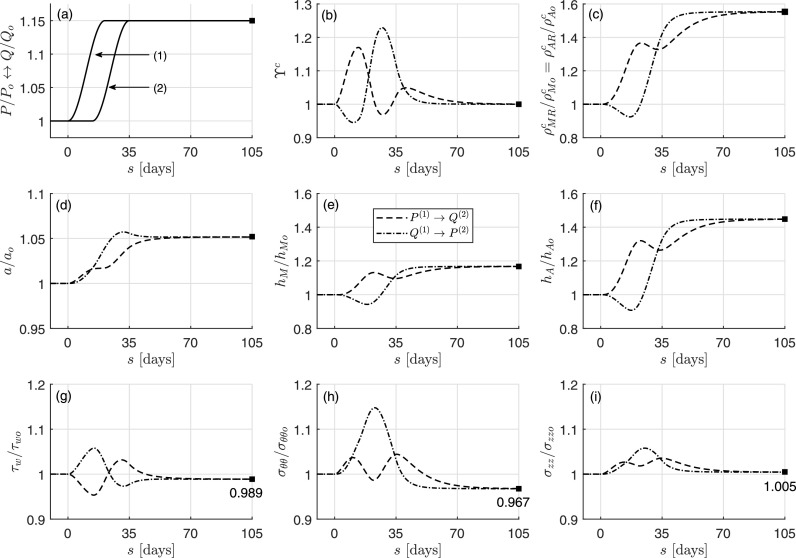
Predictions by the full constrained mixture model (first case, dashed; second case, dash-dotted) for the evolution of (c) medial and adventitial collagen (referential) mass density, (d) inner radius, (e) medial thickness, and (f) adventitial thickness, each normalized to original values, which result from two different cases of perturbations in loading [(a), solid lines] that cause (b) evolving stimulus functions (i.e., stresses different from homeostatic target values). Panels (g)–(i) show, separately, associated deviations in stress components from original homeostatic values. Note the two different time-delayed combinations of changes in pressure and flow (a). Shown, too, is the long-term, mechanobiologically equilibrated solution (solid square), which is the same for each of the two (same final) loading conditions. Note the perfect correspondence of the long-term steady-state solution computed with the present time-independent formulation and the full (hereditary) constrained mixture model.

The goal of this first example is to confirm that the rate-independent (pseudoelastic) G&R model derived in Sec. IV B can compute exactly the long-term response (Sec. IV C) of a thin-walled bilayered artery (Sec. IV A 1) when subjected to multiple external loads that are sustained for long periods. Consider, therefore, two different combinations of loading consisting of 1.15-fold increases in the applied pressure *P* and flowrate *Q*, each sustained following initial transients. To delineate better the responses to different stimuli, the loads are applied sequentially in the orders *P*^(1)^ → *Q*^(2)^ (first case) and *Q*^(1)^ → *P*^(2)^ (second case), each taking 21 days overall to reach steady values and time-shifted from the other by 14 days. See Fig. 1(a).

Panel (b) shows the resulting/evolving stimulus function ϒ^*c*^ for (both medial *M* and adventitial *A*) collagen *c* as an example. Panels (c)–(f) in Fig. 1 show the case-specific evolving responses (for $\rho_{\Gamma R}^{c}$, *a*, *h_M_*, and *h_A_*, respectively) predicted by the full model of Sec. IV A for the different combinations of loads shifted over time. Finally, fold differences in shear, circumferential, and axial stresses from homeostatic (present in ${ϒ}_{\Gamma }^{\alpha }$) are shown separately in panels (g)–(i). Note, in particular, the complex responses that are obtained by initiating the different mechanical perturbations at different times. Indeed, such simulations illustrate the importance of modeling G&R because results are not always intuitive at first given the many parallel nonlinear processes. For example, an instantaneous increase in pressure would be expected first to increase luminal radius and decrease (isochorically) the wall thickness, whereas an infinitely slow increase in pressure might result in fully adapted (quasi-equilibrium) restorations of luminal radius and increases in wall thickness at each time. Actual G&R would be expected to fall within these extremes depending on rates and extents of loading and rates of matrix turnover.

Here, because the characteristic rate of G&R (*k_G&R_* = 1/7 ≈ 0.143 days^−1^) is greater than the characteristic rate of change of the external loads (in this example, *k_ext_* ∼ 0.15/20 ≈ 0.075 days^−1^, recall Sec. IV C), both G&R processes start [Figs. 1(b)–1(f)] right after the first external load (either *P* or *Q*) increases [Fig. 1(a)]. Note, too, that the mechano-stimulus functions for collagen ϒ^*c*^ [Fig. 1(b)] and smooth muscle ϒ^*m*^ (not shown), which drive the G&R process, yield *different* short- and mid-term mass density [Fig. 1(c)] and geometric [Figs. 1(d)–1(f)] evolutions, yet regardless of the order in which the loads are applied, they yield a *common* long-term outcome at *s* = 105 days = 15*s_G&R_* ≫ *s_G&R_* = 7 days, when mechanobiological equilibrium is restored [mathematically described by ${ϒ}_{h}^{\alpha }=1$, recall Eq. (31)] and the mass production rates balance perfectly the removal rates in the evolved and now unchanging configuration. Particularly interesting is the resetting of homeostatic stresses (*τ_wh_* ≠ *τ_wo_*, *σ_θθh_* ≠ *σ_θθo_*, and *σ_zzh_* ≠ *σ_zzo_*) at the new evolved state, which, yet satisfy the more general equilibrium condition ${ϒ}_{h}^{\alpha }={ϒ}_{o}^{\alpha }=1$.

Importantly, we can also directly compute this long-term, path-independent solution using the particularized formulation of Sec. IV B, which yields a single solution for the combination of external loads γ = *P_h_*/*P_o_* = ε = *Q_h_*/*Q_o_* = 1.15, here computed via a Newton–Raphson method with initial guess given by an ideal adaptation of the type $a_{h}/a_{o}=\varepsilon^{1/3},\;h_{Mh}/h_{Mo}=h_{Ah}/h_{Ao}=\gamma \varepsilon^{1/3},\rho_{Mh}^{c}/\rho_{Mo}^{c}=1$, and $f_{zh}/f_{zo}=h_{h}(2a_{h}+h_{h})/(h_{o}(2a_{o}+h_{o}))$, see Sec. IV E. This solution is shown by solid squares in Fig. 1, which reveals precise correspondence with the full, time-dependent constrained mixture model at long (fully adapted) times.

### B. Instantaneously adapted, quasi-equilibrium, slow evolution

As discussed in Sec. IV C, rate-independent formulations derived in Sec. IV B are also valid for computing slow G&R if the characteristic rate *k_G&R_* is much greater than the characteristic rate of change of the external stimuli *k_ext_*, satisfying then the quasi-steady-state relation *k_ext_*/*k_G&R_* ≪ 1 at any G&R time *s*. For these particular situations, the arterial adaptation (occurring over a time scale *s_G&R_* = 1/*k_G&R_*) to different alterations in mechanical loading (occurring over a time scale *s_ext_* = 1/*k_ext_* ≫ 1/*k_G&R_* = *s_G&R_*) may be regarded as immediate at any G&R time *s*. We confirm this assessment in this example, quantifying at the same time how short the G&R characteristic time *s_G&R_* should be with respect to the external loading characteristic time *s_ext_* so that the predictions given by both formulations, general and particularized, become (approximately) equal. We analyze three different cases, one for each type of external perturbation that stimulates the G&R response of the artery under study (i.e., altered inner pressure, flow rate, or axial stretch), showing the different adaptive processes that the artery undergoes for each.

**FIG. 2.**
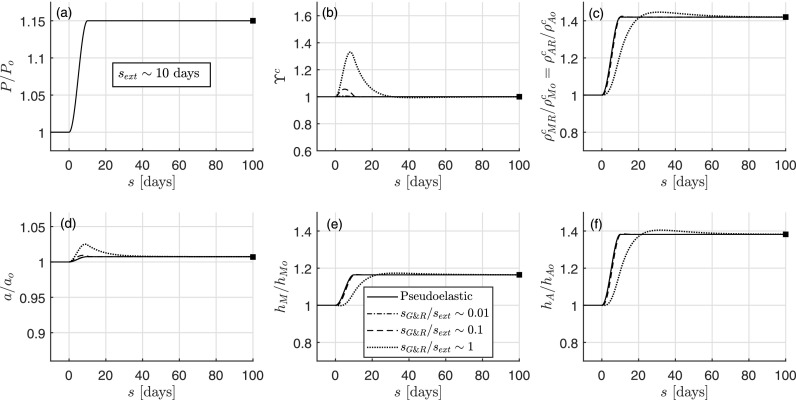
Rate-independent (solid line) and rate-dependent (non-solid lines) evolutions computed, respectively, with the pseudoelastic and the full constrained mixture models, the latter with different characteristic G&R times *s_G&R_* = {0.1, 1, 10} days, for an isolated increase in pressure with *s_ext_* ∼ 10 days. Shown are (a) prescribed load *P*/*P_o_* from 1 to 1.15, (b) mechano-stimulus function ϒ^*c*^, (c) referential mass densities of collagen $\rho_{MR}^{c}/\rho_{Mo}^{c}=\rho_{AR}^{c}/\rho_{Ao}^{c}$, (d) relative inner radius *a*/*a_o_*, (e) relative medial thickness *h_M_*/*h_Mo_*, and (f) relative adventitial thickness *h_A_*/*h_Ao_*. The final total wall thickness is *h* = 0.0494 mm (with 67% due to medial thickening and 33% due to adventitial thickening). Finally, shown too is the long-term mechanobiologically equilibrated solution (solid square), which reveals perfect correspondence of all three methods at the final adapted state. The scales are the same in Figs. 2, 3, and 4 to facilitate comparisons.

Figure 2 shows three different G&R arterial responses (non-solid lines), associated with three different G&R characteristic times *s_G&R_* = 1/*k_G&R_* = 0.1 days, 1 day, or 10 days (with $k_{G\&R}=k_{o}^{m}=k_{o}^{c}=k^{act}$), which are computed with the full model of Sec. II A for the particular increases in inner pressure *γ*(*s*) ≡ *P*(*s*)/*P_o_* shown in Fig. 2(a). In order to assess the quasi-steady-state assumption for each case, these values of *s_G&R_* are compared to the characteristic time of the external load application, which we estimate from Fig. 2(a) as the time taken for *P*/*P_o_* → 1.15, namely *s_ext_* ∼ 10 days in this case. We also show in the same figure the *single* rate-independent solution of Sec. IV B (pseudoelastic, solid line) computed as a function of the time-dependent inner pressure ratio *P*(*s*)/*P_o_* where time *s* simply plays the role of a simulation-driver parameter. In this case, we start the iterative solution procedure at each new time step using the converged solution at the previous time step.

Figure 2 shows that the prescribed increase in pressure provokes simultaneous changes in referential mass densities $\rho_{MR}^{c}$ and $\rho_{AR}^{c}$ [Fig. 2(c), with similar tendency for medial smooth muscle], inner radius *a* [Fig. 2(d)], and layer thicknesses *h_M_* and *h_A_* [Figs. 2(e) and 2(f)], driven by mechano-stimulus functions ϒ^*α*^ [Fig. 2(b) for collagen *α* = *c*, with similar tendency for smooth muscle], in four simulations (three rate-dependent based on different characteristic times *s_G&R_* and one rate-independent). In particular, the evolution of inner radius, thicknesses, and masses for *s_G&R_*/*s_ext_* ∼ 0.1/10 = 0.01, for which ϒ^*α*^ ≃ 1 [cf. Eq. (48)], is (in practice) indistinguishable from the rate-independent evolution, for which ${ϒ}_{h}^{\alpha }=1$ [cf. Eq. (31)]. For the other two simulations, *s_G&R_*/*s_ext_* ∼ 1/10 = 0.1 and *s_G&R_*/*s_ext_* ∼ 10/10 = 1, the mechano-stimulus function no longer satisfies the quasi-equilibrium condition ϒ^*α*^ ≃ 1 during early times. The evolution of luminal radius for these two cases initially separates from the mechanobiologically equilibrated solution, even though all remain close to the initial homeostatic value for our prescribed modest increase in pressure,^22^ i.e., *a*/*a_o_* ≃ 1 = *ε*^1∕3^ where *ε* = *Q*/*Q_o_*. Note, however, that the overall solution (including medial and adventitial thicknesses and constituent masses) given by the rate-independent formulation is still in good agreement with that of the full formulation. Finally, we see again that the rate-independent, all three rate-dependent, and the mechanobiologically equilibrated (solid square) simulations predict exactly the same long-term, fully equilibrated solution at *s* = 100 days, reaching a value (not shown) of the evolved total thickness *h*/*h_o_* = 1.23 which is 7% greater than that for an ideal mechanoadaptation 1.15 = *γε*^1∕3^. This 7% difference between the final value of relative total thickness (1.23) and the ideal target (1.15) can be attributed to the relatively high content of elastin within the artery under study (33% overall), which is known to prevent a perfect adaptation since elastin does not turnover.^23^ Albeit not shown, the responses of shear, circumferential, and axial over-stresses allow one to understand the transient, short-term trends in Fig. 2, as, for example, in the case of comparable timescales (*s_G&R_*/*s_ext_* ∼ 1). Indeed, the increase in pressure mainly provokes an initial increase in circumferential stress, hence the stimulus functions ϒ^*α*^ drive a growth process (with parallel increments of thickness and mass) until, eventually, stresses return close to normal values and ${ϒ}^{\alpha }\to {ϒ}_{h}^{\alpha }=1$.

**FIG. 3.**
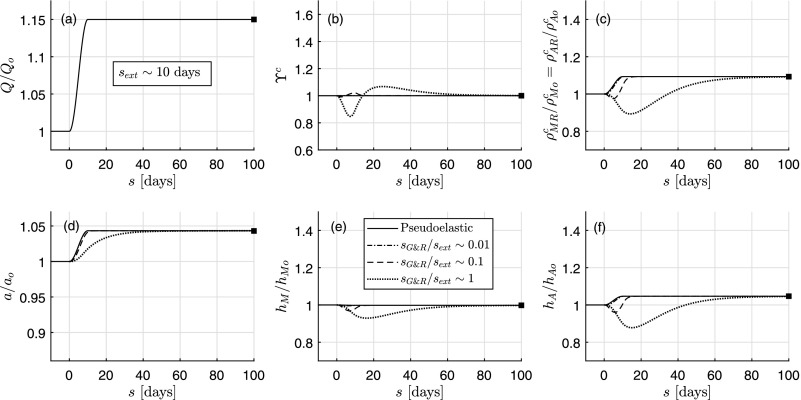
Similar to Fig. 2, except for an isolated increase in flow rate *Q* from 1 to 1.15. The final total wall thickness is *h* = 0.0407 mm (with 70% due to medial thickening and 30% due to adventitial thickening).

Figure 3 shows the same type of analysis but for a particular increase in flow rate *ε*(*s*) ≡ *Q*(*s*)/*Q_o_* [Fig. 3(a)]. We see that the rate-independent (pseudoelastic, solid line) formulation again provides a very good approximation to the quasi-static evolution predicted by the full model (case $s_{G\&R}/s_{ext}∼0.01,\;\forall s$) and exactly the same long-term, tissue-maintenance solution as the full model in all cases ($s_{G\&R}/s_{ext}∼\left\{0.01,0.1,1\right\}$, *s* = 100 days). If we compare the full model predictions for *s_G&R_*/*s_ext_* ∼ 0.1 and *s_G&R_*/*s_ext_* ∼ 1 to the single one given by the rate-independent model, we observe good agreements for the evolution of inner radius, but initially opposed tendencies for thicknesses and masses. We note that, at *s* = 100 days, $a/a_{o}=1.04≃1.05=\varepsilon^{1/3}$ and $h/h_{o}≃1.01<1.05=\gamma \varepsilon^{1/3}$ (not shown), indicative of a near but not fully mechanoadaptive solution again. In the case of comparable timescales (*s_G&R_*/*s_ext_* ∼ 1), the increase in flow rate mainly provokes an increase in shear stress, and thus a decrease in the stimulus functions ϒ^*α*^ [cf. Eq. (11)], which initially attenuates matrix turnover consistent with nitric oxide slowing collagen production by smooth muscle cells (with parallel decrements of thickness and mass). This reduced thickness, along with the increase in luminal radius (also consistent with vasodilation with nitric oxide), provokes an increase in circumferential stress that, subsequently, drives a growth process via ϒ^*α*^ > 1. Finally, stresses closely return to normal values, such that ${ϒ}^{\alpha }\to {ϒ}_{h}^{\alpha }=1$.

**FIG. 4.**
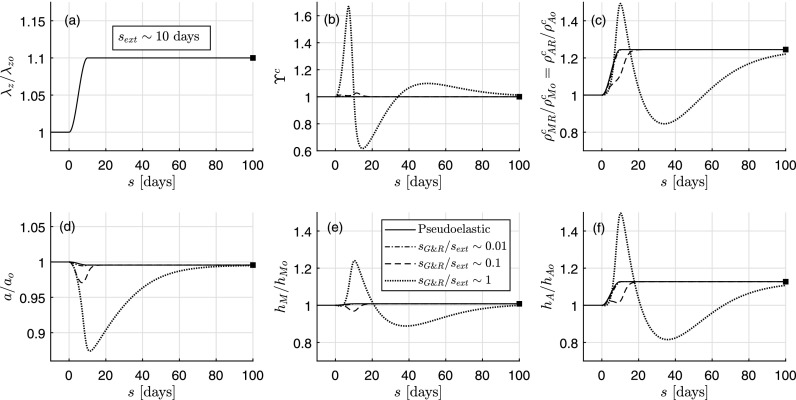
Similar to Fig. 2 except for an isolated increase in axial stretch *λ_z_* from 1 to 1.1. The final total wall thickness is *h* = 0.0419 mm (with 68% due to medial thickening and 32% due to adventitial thickening).

Similar conclusions regarding quasi-equilibrium ($s_{G\&R}/s_{ext}∼0.01,\;\forall s$) and long-term ($s_{G\&R}/s_{ext}∼\left\{0.01,0.1,1\right\}$, *s* = 100 days) predictions are obtained for a single increase in the axial stretch ratio *λ_z_*(*s*)/*λ_zo_* (in this case up to *λ_zh_*/*λ_zo_* = 1.10, Fig. 4). Regarding short- and mid-term evolutions, especially for *s_G&R_*/*s_ext_* ∼ 1, we see that all geometric and mass variable responses separate from the quasi-equilibrium solution. This finding is consistent with prior results that mechanobiological adaptations are particularly sensitive to changes in axial length.^24,25^ Finally, the common mechanobiologically equilibrated state (not fully reached at *s* = 100 days for *s_G&R_*/*s_ext_* ∼ 1) is such that *a*/*a_o_* ≃ 1 = *ε*^1∕3^ and *h*/*h_o_* = 1.05 > 1 = *γε*^1∕3^. In this case, for comparable timescales (*s_G&R_*/*s_ext_* ∼ 1), the increase in axial stretch mainly provokes an increase in axial stress and thus stimulus functions ϒ^*α*^, which initially drive a growth process (with parallel increments of thickness and mass). Yet, the substantial reduction in inner radius (because of the axial stretch) also provokes an increase in shear stress, which, subsequently, attenuates G&R via values ϒ^*α*^ < 1. Finally, because of the decreased thickness, circumferential stress increases slightly, driving G&R until the artery reaches a mechanobiologically equilibrated state associated with ${ϒ}^{\alpha }\to {ϒ}_{h}^{\alpha }=1$. Interestingly, the mechano-stimulus functions ϒ^*α*^ [panel (b) in Figs. 2–4 for the specific case of collagen] go through one extremum only (i.e., a maximum) in the case of an isolated increase in pressure, two extrema (i.e., a minimum and a maximum) in the case of an isolated increase in flow rate, and three extrema (i.e., a maximum, a minimum, and a last maximum) in the case of an isolated increase in axial stretch, which, in any case, equal the number of primary mechanical stimuli that are being stimulated sequentially.

**FIG. 5.**
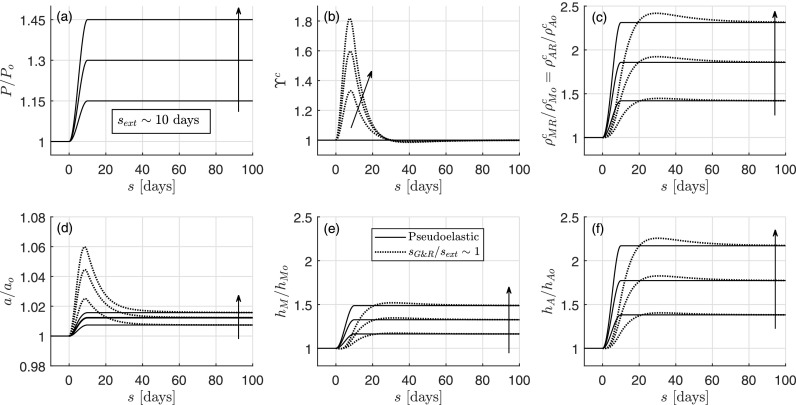
Rate-independent (solid lines) and rate-dependent (dotted lines) evolutions computed, respectively, with the pseudoelastic and the full constrained mixture models, the latter with a single characteristic G&R time *s_G&R_* ∼ 10 days, for different increases in pressure with *s_ext_* ∼ 10 days. Shown are (a) prescribed loads *P*/*P_o_* from 1 to 1.15, 1.30, and 1.45, (b) respective mechano-stimulus functions ϒ^*c*^, (c) referential mass densities of collagen $\rho_{MR}^{c}/\rho_{Mo}^{c}=\rho_{AR}^{c}/\rho_{Ao}^{c}$, (d) relative inner radius *a*/*a_o_*, (e) relative medial thickness *h_M_*/*h_Mo_*, and (f) relative adventitial thickness *h_A_*/*h_Ao_*.

Finally, Fig. 5 shows results similar to those in Fig. 2 except for three different degrees of increased luminal pressure *γ*(*s*) = *P*(*s*)/*P_o_* for the slowest response examined in Fig. 2 (*s_G&R_*/*s_ext_* ∼ 1). As it can be seen, increases in the perturbation in pressure from 1.15 to 1.30 to 1.45 fold results in progressively slower adaptations as expected. Nevertheless, in each case, the pseudoelastic model (solid curves) provides the same long-term predictions as the full model (dotted curves) and even provides reasonable predictions over the short-term in some metrics.

## III. DISCUSSION

Biological growth (changes in mass) and remodeling (changes in microstructure) processes are necessarily time dependent due to the finite periods needed for the significant material to be synthesized, deposited, degraded, and/or reorganized. Moreover, rates of degradation appear to depend in part on the conditions present at the time of deposition (e.g., how the constituent was oriented or cross-linked), hence endowing tissues with a “material memory.” For this reason, hereditary integral formulations, as commonly used in viscoelasticity, have proven useful in describing and predicting evolving geometries, compositions, and properties of soft tissues under diverse conditions.^3–9^

Nevertheless, such formulations can be computationally expensive and there is strong motivation to identify additional methods for analysis as well as the conditions for which such methods hold. In this paper, we presented a time-independent (“pseudoelastic”) formulation of arterial G&R to describe particular behaviors of otherwise time-dependent processes, which parallels Fung's use of pseudoelasticity to describe particular behaviors of otherwise viscoelastic tissues. We show that this approach, represented by a system of nonlinear algebraic equations for a bilayered, thin-walled artery, yields the same outcomes as a general constrained mixture model, represented by a system of nonlinear integro-differential equations, for both long-term steady state responses in which the external loads are sustained over time^13^ and quasi-equilibrium evolutions in which the external loads change slowly enough that the arterial wall can essentially adapt instantaneously to the given alterations at each G&R time *s*. In the latter case, the quasi-equilibrium formulation gives good qualitative results even for some cases in which rate-dependent effects remain, especially for isolated increases in blood pressure. This last observation, along with a marked reduction in computational time, makes the present pseudoelastic G&R formulation a good starting point for studying more complex situations that necessitate a general solution given by the full integral model. Importantly, the time needed to compute the rate-independent formulation (in evolution form) was ∼10 to 100 times less than that for the full model (when run in MATLAB on a 12 dual-core processor, and depending on specific values of *s_G&R_*, which affect the time step of the integral formulation).

Obviously, even though (rate-independent) pseudoelasticity will never replace (rate-dependent) viscoelasticity, hyperelastic models describing the pseudoelastic behavior of viscoelastic tissues continue to play important roles during stages of experimental characterization, the computation of important responses, both analytically and numerically, the delineation of limiting responses, and in straightforward determinations of long-term outcomes following relaxation and creep.^15,26–28^ In parallel, time-independent G&R models as presented herein will never replace time-dependent G&R models, either fully integrated^2^ or kinematically motivated.^29^ We submit, however, that pseudoelastic G&R formulations can play parallel roles as in Fung's pseudoelasticity to better understand complex G&R of soft tissues, in general, and of arteries, in particular. Furthermore, because full^2^ and mechanobiologically equilibrated^13^ formulations predict the same long-term steady state outcomes, these simplified formulations can serve as efficient tools for constrained mixture modeling that includes studies of parameter sensitivity, uncertainty quantification, and optimization, which tend to be even more demanding computationally. Regarding material characterization of evolving tissues, the present formulation can be used, for example, to determine G&R-related material parameters by comparing original homeostatic and evolved homeostatic configurations [via, for example, Eqs. (39) and (40)], hence simplifying this important stage of constitutive modeling. Finally, this type of modeling G&R could also guide the process of engineering new tissues that react to artificially induced mechanical loading, as in Refs. 30–32.

For purposes of illustration, we included characteristic times (half-lives) for G&R that may be regarded unrealistic except in cases of extreme adaptations or pathologies (e.g., *s_G&R_* ∼ 0.1 days), which we used as a more stringent test of the concept. Nevertheless, values of *s_G&R_* should be assessed relative to a characteristic time of changes in the loads that stimulate G&R, namely *s_ext_*. Thus, a dimensionless number of the type *s_G&R_*/*s_ext_*, comparing characteristic times of G&R and loading, is what actually determines the goodness of a quasi-static assumption. Regarding actual arterial behaviors, *s_G&R_* may range from ∼10 to 100 days^17,33^ whereas changes in loading may range from seconds to many days,^21,34^ hence dimensionless numbers *s_G&R_*/*s_ext_* of order unity, or lower, exist for *s_ext_* ∼10 to 100 days or longer. The lower the value of *s_G&R_*/*s_ext_*, the better the pseudoelastic G&R approximation, as one would expect from viscoelasticity theory. In contrast, the greater the ratio *s_G&R_*/*s_ext_*, the better the transient “elastic” (without G&R) approximation; see Example 2 in Ref. 13. Because *s_G&R_* is material-dependent, it can change during certain conditions or diseases,^33^ hence each situation should be evaluated individually.

Timescales for G&R and mechanical loading can be very different in other situations, but analyses in terms of orders of magnitude are yet useful.^35^ Indeed, kinematic models of growth^29^ ultimately rely on the recognition of these two time scales, with evolution of the growth tensor **F**_*g*_ depending on the “growth (or) remodeling timescale.”^35^ Hence, growth laws may be expressed in kinematic models in terms of rate parameters^36^ that, alternatively, may depend on problem-specific growth metrics to prevent unlimited growth,^37^ with baseline values identified as rates of initial growth.^38^ Similarly, the so-called global growth approach^39^ also postulates evolution equations for the stress-free state of an artery, where, again, characteristic times associated with the growth process can be identified.

In conclusion, we emphasize that all of the illustrative results (Figs. 1–5) also depend on all of the particular constitutive assumptions, including functional forms (Secs. IV A and IV E) and prescribed values (Table I). Indeed, whereas we must continue to search for mechanobiologically appropriate and yet computationally tractable theoretical frameworks, the primary need—as noted by Fung decades ago—remains identification of the best constitutive relations, which for constrained mixture theories of G&R includes descriptors of mechanical behaviors, rates of mass production, mechanisms of incorporation within extant matrix, and rates of mass removal for each structurally significant constituent. Improved relations for complex pathologies representing maladaptation remain wanting. Theoretically motivated experiments are thus essential, which as we show herein should begin to focus more on the time scales over which loading and biological processes occur.

**TABLE I.** Baseline material parameters for a mouse descending thoracic aorta. Best-fit values determined from Ref. 21, and adapted, for the specific examples performed in this work. In particular, $\left[k_{o}^{m},k_{o}^{c}\right]$ values would typically be of order 1/70 days^−1^ under normal conditions,^17^ but we consider here a faster rate of turnover^33^ as a more stringent test for the pseudoelastic G&R framework. Superscripts *e*, *m*, and *c* denote elastin, smooth muscle, and collagen, with superscripts/subscripts *r*, *θ*, *z*, and *d* denoting radial, circumferential, axial, and symmetric diagonal directions. Subscripts *M* and *A* denote medial and adventitial layers whereas *o* denotes original homeostatic values. Subscripts *σ* and *τ* denote intramural and wall shear stresses, respectively. See Table II for definitions of other variables.

Artery mass density	*ρ*	1050 kg/m^3^
Medial mass fractions	$\left[\phi_{Mo}^{e},\phi_{Mo}^{m,\theta },\phi_{Mo}^{c,z},\phi_{Mo}^{c,d}\right]$	[0.4714, 0.4714, 0.0381, 0.0190]
Adventitial mass fractions	$\left[\phi_{Ao}^{e},\phi_{Ao}^{c,\theta },\phi_{Ao}^{c,z},\phi_{Ao}^{c,d}\right]$	[0.0333, 0.0175, 0.3201, 0.6291]
Diagonal collagen orientation	*α*_0_	45.36°
Inner radius, thicknesses	[*a_o_*, *h_Mo_*, *h_Ao_*]	[0.6468, 0.0284, 0.0118] mm
Elastin material parameter	*c^e^*	114.5 kPa
Smooth muscle parameters	$\left[c_{1}^{m},c_{2}^{m}\right]$	[401.0 kPa, 0.012]
Collagen parameters	$\left[c_{1}^{c},c_{2}^{c}\right]$	[411.2 kPa, 5.5]
Deposition stretches	$\left[G_{r}^{e},G_{\theta }^{e},G_{z}^{e}\right]$	[1/(1.9 × 1.6), 1.9, 1.6]
Deposition stretches	$\left[G_{\theta }^{m}=G_{\theta }^{c},G_{z}^{c},G_{d}^{c}\right]$	[1.071, 1.193, 1.192]
Maximum active stress	*T*_max_	100 kPa
Active stretch limits	[*λ_M_*, *λ*_0_]	[1.1, 0.4]
Vasoactive parameters	[*C_B_*, *C_S_*]	0.8326× [1, 0.5]
Vasoactive rate parameter	*k^act^*	1/7 day^−1^
Mass production gains	$\left[K_{\sigma }^{m},K_{\tau }^{m},K_{\sigma }^{c},K_{\tau }^{c}\right]$	[1.6, 2, 2, 2.5]
Mass removal rates	$\left[k_{o}^{m},k_{o}^{c}\right]$	[1/7, 1/7] day^−1^

## IV. METHODS

### A bilayered constrained mixture model for arteries

A.

First consider the kinematics and evolution equations of a constrained mixture model^2^ that governs G&R processes of an idealized bilayered artery that comprises a medial and an adventitial layer. Each layer is modelled as an independent constrained mixture having its own *local* variables that must satisfy kinematic compatibility constraints at the medial-adventitial interface. This time-dependent, integral-type formulation will be particularized in Sec. IV B to special cases for which the time dependence either vanishes or can be neglected, following ideas introduced in Ref. 13.

#### 1. Geometry, mass fractions, and kinematics

**FIG. 6.**
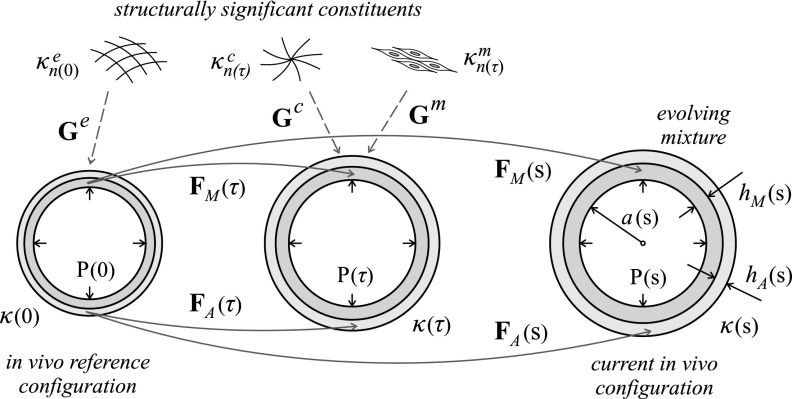
Evolving *in vivo* configurations of a bilayered arterial wall from time *s* = 0 (original homeostatic reference configuration *κ*(0) ≡ *κ_o_*) to an arbitrary time *s* > 0 [current configuration *κ*(*s*)], showing the different deformation gradients **F**_*M*_ and **F**_*A*_ experienced by the medial and adventitial layers over time, in particular at times 0 ≤ *τ* ≤ *s*. Shown, also, are the different natural configurations $\kappa_{n\left(\tau \right)}^{\alpha }$ for the different structurally significant constituents *α* (elastin “*e*,” smooth muscle “*m*,” and collagen “*c*”), which are deposited with separate but constant deposition stretches **G**^*α*^ within both layers at the indicated deposition times *τ* (except for the smooth muscle, present in media only), as well as geometric parameters *a* (luminal radius), *h_M_* (medial thickness), and *h_A_* (adventitial thickness) and luminal pressure *P*, which can also change over time.

Figure 6 illustrates an idealized bilayered cross-section of a normal elastic artery wherein the media (inner layer, since the normal intima is not expected to carry significant loads) is the functional layer and the adventitia (outer layer) bears increasingly more load as the pressure increases abruptly.^16^ Three illustrative *in vivo* configurations are shown at three respective G&R times (on orders of days to weeks). Let the (original) homeostatic configuration at time *s* = 0, namely *κ_o_* = *κ*(0), serve as a reference. The configuration at the current G&R time *s* > 0 is denoted *κ*(*s*), while configurations at all intermediate times 0 ≤ *τ* ≤ *s* are denoted by *κ*(*τ*). Hereafter, for the sake of notational simplicity, we will omit the time dependence when it is not needed explicitly. We denote the inner (luminal) radius of the artery in a generic configuration as *a*, the medial thickness as *h_M_*, and the adventitial thickness as *h_A_*. The total wall thickness is thus *h *=* h_M_* + *h_A_*. The length of the artery is *l*, which is the same for both layers by compatibility of axial displacements at the medial-adventitial interface *r_MA_* = *a *+* h_M_*, hence *l_M_* = *l_A_* ≡ *l*.

We analyze the wall mechanics using a thin-walled approach in which variables within each layer are uniform, though different, consistent with effects of residual stress [Residual stresses tend to homogenize transmural distributions of stress (Refs. 16 and 17), thus rendering this assumption reasonable]. Hence, we separately compute the deformation gradient tensor for each layer, which quantifies motions between the original homeostatic reference configuration *κ_o_* and a generic configuration *κ*. Using cylindrical coordinates *X_rθz_* = {*r*, *θ*, *z*} to denote the position of a material point within either the media (*M*) or the adventitia (*A*), and assuming that cylindrical axes coincide with principal directions of strain, the respective layer-specific deformation gradients read

$${\left[F_{M}\right]}_{r\theta z}=\text{diag}[\lambda_{Mr},\lambda_{M\theta },\lambda_{Mz}],$$ (1)

and

$${\left[F_{A}\right]}_{r\theta z}=\text{diag}[\lambda_{Ar},\lambda_{A\theta },\lambda_{Az}],$$ (2)

with stretches

$$\lambda_{Mr}=\frac{h_{M}}{h_{Mo}},\;\lambda_{M\theta }=\frac{a+h_{M}/2}{a_{o}+h_{Mo}/2},\;\lambda_{Mz}=\lambda_{z}\equiv \frac{l}{l_{o}},$$ (3)

and

$$\lambda_{Ar}=\frac{h_{A}}{h_{Ao}},\;\lambda_{A\theta }=\frac{r_{MA}+h_{A}/2}{r_{MAo}+h_{Ao}/2},\;\lambda_{Az}=\lambda_{z}\equiv \frac{l}{l_{o}},$$ (4)

where *r_MA_* = *a *+* h_M_* enforces compatibility of radial (and circumferential) displacements at the medial-adventitial interface. Subscript *o* refers to the original homeostatic state.

The material composition is also layer-specific. In particular, we consider three primary types of load-bearing constituents (elastin-dominated “*e*,” smooth muscle “*m*,” and fibrillar collagen-dominated “*c*”) , with mass fractions $\phi_{M}=[\phi_{M}^{e},\phi_{M}^{m},\phi_{M}^{c}]$ satisfying $\sum_{\alpha }^{e,m,c}\phi_{M}^{\alpha }=1$ in the media, and $\phi_{A}=[\phi_{A}^{e},\phi_{A}^{m},\phi_{A}^{c}]$ satisfying $\sum_{\alpha }^{e,m,c}\phi_{A}^{\alpha }=1$ in the adventitia, with $\phi_{A}^{m}=0$.^17^ Following a constrained mixture approach for each layer, the motion of each constituent (and cohort thereof) is constrained to equal the motion of the corresponding layer as a whole, as given by deformation gradients **F**_*M*_ and **F**_*A*_ in Eqs. (1) and (2). Each constituent and cohort, however, has its own evolving natural configuration $\kappa_{n}^{\alpha }\left(\tau \right)$, where *τ* denotes the instant at which that constituent mass was produced and deposited within the arterial wall (see Fig. 6). Without a loss of generality, we let the different constituents deposit in the media and adventitia with layer-independent deposition (symmetric) stretch tensors **G**^*α*^, which means that the natural configuration of a constituent *α* at time *τ*, namely, $\kappa_{n}^{\alpha }\left(\tau \right)$, is common for both layers (see Fig. 6). In addition, we assume (in physiologic adaptations) constant and volume-preserving deposition stretch tensors **G**^*α*^, with $\det G^{\alpha }=1$. Following the deformation path shown in Fig. 6, the deformation gradient $F_{\Gamma n\left(\tau \right)}^{\alpha }\left(s\right)$ experienced by constituent *α* deposited at time *τ* within layer Γ = *M*, *A* (when applicable) that survives to current G&R time *s* reads for smooth muscle and collagen, which are continuously produced and removed, as^4^

$$F_{\Gamma n\left(\tau \right)}^{\alpha }\left(s\right)=F_{\Gamma }\left(s\right)F_{\Gamma }^{-1}\left(\tau \right)G^{\alpha },\;\alpha =m,c,$$ (5)

and for elastin, which is produced perinatally and thus at *s* ≤ 0 in our case of G&R in maturity,^6^ as

$$F_{\Gamma }^{\alpha }\left(s\right):=F_{\Gamma n\left(0\right)}^{\alpha }\left(s\right)=F_{\Gamma }\left(s\right)G^{\alpha },\;\alpha =e,$$ (6)

since **F**_Γ_(*τ* = 0) = **I**.

#### 2. Mass and strain energy evolutions

Smooth muscle cells and collagen fibers are produced continuously, hence their removal is usually accounted for in constrained mixtures models through mass survival (exponential decay) functions $q_{\Gamma }^{\alpha }\left(s,\tau \right)\in [0,1]$ of the type (we use the form introduced in Ref. 13, but others are possible^18^)

$$q_{\Gamma }^{\alpha }\left(s,\tau \right)=\text{exp}\;\left(-\int_{\tau }^{s}k_{\Gamma }^{\alpha }\left(t\right)dt\right),\;\alpha =m,c,$$ (7)

where the rate “parameter” $k_{\Gamma }^{\alpha }\left(t\right)$, with *τ* ≤ *t *≤* s*, is assumed (constitutively) to increase with respect to its original homeostatic constant value $k_{\Gamma o}^{\alpha }$ through

$$k_{\Gamma }^{\alpha }\left(t\right)=k_{\Gamma o}^{\alpha }(1+{\left(\Delta \sigma \left(t\right)\right)}^{2}),\;\alpha =m,c,$$ (8)

with Δ*σ*(*t*) quantifying any relative difference between a given scalar measure of intramural Cauchy stress (e.g., magnitude, principal invariant, maximum principal value) acting at time *t* at the tissue level, namely $\tilde{\sigma }\left(t\right)$, and its corresponding homeostatic value ${\tilde{\sigma }}_{o}$, such that

$$\Delta \sigma \left(t\right)=\frac{\tilde{\sigma }\left(t\right)-{\tilde{\sigma }}_{o}}{{\tilde{\sigma }}_{o}}\;.$$ (9)

Additionally, production of collagen and smooth muscle is governed constitutively by respective mass density production relations. Following Ref. 13 for the mass production rate of cohort *α* per unit reference volume of the mixture (in this case, within layer Γ), we have for both layers (see Tables I and II for nomenclature)

$$m_{\Gamma R}^{\alpha }\left(\tau \right)=m_{\Gamma N}^{\alpha }\left(\tau \right){ϒ}_{\Gamma }^{\alpha }\left(\tau \right)=k_{\Gamma }^{\alpha }\left(\tau \right)\rho_{\Gamma R}^{\alpha }\left(\tau \right){ϒ}_{\Gamma }^{\alpha }\left(\tau \right),$$ (10)

where $m_{\Gamma N}^{\alpha }\left(\tau \right)=k_{\Gamma }^{\alpha }\left(\tau \right)\rho_{\Gamma R}^{\alpha }\left(\tau \right)>0$ is an evolving *nominal* mass production rate including, importantly, the same function $k_{\Gamma }^{\alpha }\left(\tau \right)$ employed for mass removal and the referential mass density $\rho_{\Gamma R}^{\alpha }\left(\tau \right)$ of constituent *α* within layer Γ (i.e., per unit reference volume of the respective layer Γ). This relation ensures balanced production and removal in homeostatic states,^13^ for which $m_{\Gamma R}^{\alpha }\to m_{\Gamma N}^{\alpha }$. Moreover, ${ϒ}_{\Gamma }^{\alpha }\left(\tau \right)$ is a “stimulus function” that ultimately drives mass production to rates different from nominal depending on biochemomechanical stimuli; herein, we consider mechano-stimuli only. Over the years, we have found that a reasonable form for ${ϒ}_{\Gamma }^{\alpha }\left(\tau \right)$ for arteries subjected to perturbed blood pressures and flows, linearized about the initial homeostatic state, reads^4^

$${ϒ}_{\Gamma }^{\alpha }\left(\tau \right)=1+K_{\Gamma \sigma }^{\alpha }\Delta \sigma \left(\tau \right)-K_{\Gamma \tau }^{\alpha }\Delta \tau_{w}\left(\tau \right),$$ (11)

where $K_{\Gamma \sigma }^{\alpha }$ and $K_{\Gamma \tau }^{\alpha }$ are constituent- and layer-specific (constant) gain parameters, *τ_w_* is the flow-induced shear stress acting over the endothelium, and Δ*τ_w_* = (*τ_w_* – *τ_wo_*)/*τ_wo_*. In particular, ${ϒ}_{\Gamma o}^{\alpha }=1$ at the original homeostatic state, where Δ*σ* = 0 = Δ*τ_w_*.

**TABLE II.** Definition of volume-specific variables. MPR, Mass Production Rate; SEF, Strain Energy Function; *α* = {*e*, *m*, *c*}, Γ = {*M*, *A*}.

Mass of constituent *α* within layer Γ per unit current volume of layer Γ	$\rho_{\Gamma }^{\alpha }$
Mass of constituent *α* within layer Γ per unit reference volume of layer Γ	$\rho_{\Gamma R}^{\alpha }$
MPR of constituent *α* within layer Γ per unit reference volume of layer Γ	$m_{\Gamma R}^{\alpha }$
Nominal MPR of constituent *α* within layer Γ per unit reference volume of layer Γ	$m_{\Gamma N}^{\alpha }$
Volume-specific SEF of constituent *α* (defined at the constitutive level)	${\hat{W}}^{\alpha }$
SEF of constituent *α* within layer Γ per unit reference volume of layer Γ	$W_{\Gamma R}^{\alpha }$

Given constitutive equations for production and removal, the evolution of the layer-specific referential mass density of the cohort *α* is^4,13^

$$\rho_{\Gamma R}^{\alpha }\left(s\right)=\int_{-\infty }^{s}m_{\Gamma R}^{\alpha }\left(\tau \right)q_{\Gamma }^{\alpha }\left(s,\tau \right)d\tau .$$ (12)

Assuming that the spatial mass density *ρ* of the arterial wall (i.e., its current mass per unit current volume of mixture) remains constant, the strain energy function of constituent *α* within layer Γ, also defined per unit reference volume of layer Γ, reads^13^

$$W_{\Gamma R}^{\alpha }(s)=\frac{1}{\rho }\int_{-\infty }^{s}m_{\Gamma R}^{\alpha }\left(\tau \right)q_{\Gamma }^{\alpha }\left(s,\tau \right){\hat{W}}^{\alpha }(C_{\Gamma n\left(\tau \right)}^{\alpha }(s))d\tau$$ (13)

where ${\hat{W}}^{\alpha }(C_{\Gamma n\left(\tau \right)}^{\alpha }\left(s\right))$ is the volume-specific strain energy function of constituent *α* and $C_{\Gamma n\left(\tau \right)}^{\alpha }\left(s\right)$ is the right Cauchy–Green deformation tensor obtained from $F_{\Gamma n\left(\tau \right)}^{\alpha }\left(s\right)$, which for smooth muscle and collagen reads

$$C_{\Gamma n\left(\tau \right)}^{\alpha }\left(s\right)=F_{\Gamma n\left(\tau \right)}^{\alpha T}\left(s\right)F_{\Gamma n\left(\tau \right)}^{\alpha }\left(s\right)=G^{\alpha }F_{\Gamma }^{-T}\left(\tau \right)C_{\Gamma }\left(s\right)F_{\Gamma }^{-1}\left(\tau \right)G^{\alpha },$$ (14)

and for elastin, reads

$$C_{\Gamma }^{e}\left(s\right):=C_{\Gamma n\left(0\right)}^{e}\left(s\right)=F_{\Gamma }^{eT}\left(s\right)F_{\Gamma }^{e}\left(s\right)=G^{e}C_{\Gamma }\left(s\right)G^{e}\;,$$ (15)

with $C_{\Gamma }=F_{\Gamma }^{T}F_{\Gamma }$. Since *ρ* remains constant

$$\rho =\sum_{\alpha }^{e,m,c}\rho_{\Gamma }^{\alpha }\left(\tau \right),\;\forall \tau ,\;\Gamma =M,A,$$ (16)

with $\rho_{\Gamma }^{\alpha }$ representing the current mass density of constituent *α* within layer Γ (i.e., per unit current volume of the respective layer Γ at each time *τ*).

#### 3. Passive and active stresses

The mechanical response of an artery is assumed to be isochoric for transient deformations at each fixed G&R time *s*. The layer-specific Cauchy stress tensor thus reads

$$\sigma_{M}\left(s\right)=\sum_{\alpha }^{e,m,c}\sigma_{M}^{\alpha }\left(s\right)+\sigma^{act}\left(s\right)-p_{M}\left(s\right)I\;,$$ (17)

in the media, and

$$\sigma_{A}\left(s\right)=\sum_{\alpha }^{e,c}\sigma_{A}^{\alpha }\left(s\right)-p_{A}\left(s\right)I\;,$$ (18)

in the adventitia, where $\sigma_{\Gamma }^{\alpha }$ is the deformation-dependent part^5^ of the Cauchy stress for constituent *α* within layer Γ, $\sigma^{act}$ is the active stress tensor generated by the smooth muscle within the media, and *p*_Γ_ are layer-specific pressure-type Lagrange multipliers associated with the incompressibility constraints $J_{M}=\det\left(F_{M}\right)$ and $J_{A}=\det\left(F_{A}\right)$ (transiently constant) that are to be determined from equilibrium and boundary conditions at fixed G&R times.

The passive Cauchy stresses for collagen and smooth muscle can be obtained from their associated second Piola–Kirchhoff stresses, deriving from the layer-specific strain energy functions $W_{\Gamma R}^{\alpha }$ of Eq. (13)^13^

$$S_{\Gamma }^{\alpha }\left(s\right)=2\frac{\partial W_{\Gamma R}^{\alpha }(s)}{\partial C_{\Gamma }(s)}=\frac{1}{\rho }\int_{-\infty }^{s}m_{\Gamma R}^{\alpha }\left(\tau \right)q_{\Gamma }^{\alpha }\left(s,\tau \right)F_{\Gamma }^{-1}\left(\tau \right)G^{\alpha }{\hat{S}}_{\Gamma }^{\alpha }(C_{\Gamma n\left(\tau \right)}^{\alpha }\left(s\right))G^{\alpha }F_{\Gamma }^{-T}\left(\tau \right)d\tau ,$$ (19)

with the second Piola–Kirchhoff stress tensor at the constituent level, deriving from ${\hat{W}}^{\alpha }(C_{\Gamma n\left(\tau \right)}^{\alpha }(s))$, as

$${\hat{S}}_{\Gamma }^{\alpha }(C_{\Gamma n\left(\tau \right)}^{\alpha }\left(s\right))=2\frac{\partial {\hat{W}}^{\alpha }(C_{\Gamma n\left(\tau \right)}^{\alpha }\left(s\right))}{\partial C_{\Gamma n\left(\tau \right)}^{\alpha }\left(s\right)}.$$ (20)

The push-forward operation over $S_{\Gamma }^{\alpha }\left(s\right)$

$$\sigma_{\Gamma }^{\alpha }\left(s\right)=\frac{1}{J_{\Gamma }\left(s\right)}F_{\Gamma }\left(s\right)S_{\Gamma }^{\alpha }\left(s\right)F_{\Gamma }^{T}\left(s\right),$$ (21)

yields the respective Cauchy stress tensor $\sigma_{\Gamma }^{\alpha }\left(s\right)$ to be used further in Eqs. (17) or (18).

If we consider that elastin is neither produced nor degraded for *s* > 0 (because functional elastin is produced during the perinatal period, and elastin tends to degrade very slowly except in pathologies characterized by marked proteolytic activity^19^) then $\rho_{\Gamma R}^{e}\left(s\right)=\rho_{\Gamma R}^{e}\left(0\right)=:\rho_{\Gamma R}^{e}$ and the strain energy function for elastin is

$$W_{\Gamma R}^{e}(s)=\frac{\rho_{\Gamma R}^{e}}{\rho }{\hat{W}}^{e}(C_{\Gamma }^{e}\left(s\right))\;.$$ (22)

The resulting second Piola–Kirchhoff stresses are

$$S_{\Gamma }^{e}\left(s\right)=2\frac{\partial W_{\Gamma R}^{e}(s)}{\partial C_{\Gamma }(s)}=\frac{\rho_{R}^{e}}{\rho }G^{e}{\hat{S}}_{\Gamma }^{e}(C_{\Gamma }^{e}\left(s\right))G^{e},$$ (23)

where ${\hat{S}}_{\Gamma }^{e}=2\partial {\hat{W}}^{e}(C_{\Gamma }^{e})/\partial C_{\Gamma }^{e}$ represents the layer-specific stress tensor at the constituent level. The passive Cauchy stresses in Eqs. (17) or (18) are obtained from Eq. (21), with *α* = *e*.

The active tensile stress generated by the smooth muscle tone within the media is considered to be exerted along the circumferential direction **e**_*θ*_, namely,^3^

$$\sigma^{act}\left(s\right)=\phi_{M}^{m}\left(s\right)T_{\max}\left(1-e^{-C^{2}\left(s\right)}\right)\lambda_{\theta }^{m\left(act\right)}\left(s\right)\left[1-{\left(\frac{\lambda_{M}-\lambda_{\theta }^{m\left(act\right)}\left(s\right)}{\lambda_{M}-\lambda_{0}}\right)}^{2}\right]e_{\theta }⊗e_{\theta },$$ (24)

where $\phi_{M}^{m}\left(s\right)=\rho_{M}^{m}\left(s\right)/\rho$ is the spatial mass fraction, $T_{\max}$ is the maximum stress that the muscle can generate, *C*(*s*) > 0 is a ratio of vasoconstrictors (e.g., endothelin-1) to vasodilators (e.g., nitric oxide), *λ_M_* and *λ*_0_ are the stretches at which the active force generating capability either is maximum or vanishes, respectively, and $\lambda_{\theta }^{m\left(act\right)}\left(s\right)$ is the current active muscle fiber stretch. The ratio *C*(*s*) is written in terms of wall shear stress through^3^

$$C\left(s\right)=C_{B}-C_{S}\Delta \tau_{w}\left(s\right),$$ (25)

where *C_B_* is a basal ratio and *C_S_* is a scaling factor, noting that vasodilators are produced by the endothelium when Δ*τ_w_* > 0 and vasoconstrictors are produced when Δ*τ_w_* < 0. Finally, the circumferential stretch for the active tone is defined as $\lambda_{\theta }^{m\left(act\right)}\left(s\right)=a\left(s\right)/a^{act}\left(s\right)$, with *a*(*s*) being the current luminal radius and *a^act^*(*s*) being an active reference length whose evolution may be modeled through^3^

$$\frac{da^{act}\left(s\right)}{ds}=k^{act}\left(a\left(s\right)-a^{act}\left(s\right)\right),$$ (26)

where 1/*k^act^* is a characteristic time of remodeling and *a^act^*(0) = *a*(0). An integral-type (convolution) solution of Eq. (26) for *a^act^*(*s*) reads

$$a^{act}\left(s\right)=\int_{-\infty }^{s}k^{act}a\left(\bar{\tau }\right)e^{-k^{act}(s-\bar{\tau })}d\bar{\tau }\;.$$ (27)

### B. Rate-independent solution for given pressure, flow rate, and axial stretch

We now recognize that the hereditary integral formulation accounts primarily for the finite half-lives of the cells and matrix that were incorporated within the evolving tissue at different past times. If, however, the tissue adapts fully to the prior perturbation in loading and all constituents produced during the adaptive period have been replaced with new constituents (continuously) produced in the new homeostatic state, then we can pre-integrate the prior, time-dependent G&R formulation following Ref. 13. In doing so, we obtain an associated time-independent solution for the bilayered G&R model outlined above. As we show below, this particularized formulation yields a system of algebraic equations that can be solved efficiently and whose solution represents either a steady-state, long-term solution reached at a G&R time *s* much greater than a characteristic time *s_G&R_* over which the G&R processes take part,^13^ namely, at *s *≫* s_G&R_*, or more importantly a quasi-equilibrium G&R evolution at any time *s* (in this case, *s* playing the role of a parameter), as we explain in Sec. IV C.

Substitution of Eq. (10) into Eq. (12) yields

$$\rho_{\Gamma R}^{\alpha }\left(s\right)=\int_{-\infty }^{s}k_{\Gamma }^{\alpha }\left(\tau \right)\rho_{\Gamma R}^{\alpha }\left(\tau \right){ϒ}_{\Gamma }^{\alpha }\left(\tau \right)q_{\Gamma }^{\alpha }\left(s,\tau \right)d\tau ,$$ (28)

which can be integrated for constant (i.e., fully evolved homeostatic, denoted by subscript *h*) values at times *s *≫* s_G&R_*, with $k_{\Gamma }^{\alpha }\to k_{\Gamma h}^{\alpha },\;\rho_{\Gamma R}^{\alpha }\to \rho_{\Gamma Rh}^{\alpha }$ and ${ϒ}_{\Gamma }^{\alpha }\to {ϒ}_{\Gamma h}^{\alpha }$, to give

$$\rho_{\Gamma Rh}^{\alpha }=k_{\Gamma h}^{\alpha }\rho_{\Gamma Rh}^{\alpha }{ϒ}_{\Gamma h}^{\alpha }\int_{-\infty }^{s}q_{\Gamma h}^{\alpha }\left(s,\tau \right)d\tau =\rho_{\Gamma Rh}^{\alpha }{ϒ}_{\Gamma h}^{\alpha },$$ (29)

since the integral of $q_{\Gamma }^{\alpha }\left(s,\tau \right)\to q_{\Gamma h}^{\alpha }\left(s,\tau \right)$, as given in Eq. (7) with constant $k_{\Gamma h}^{\alpha }$, is

$$\int_{-\infty }^{s}q_{\Gamma h}^{\alpha }\left(s,\tau \right)d\tau =\int_{-\infty }^{s}\;\text{exp}\;(-k_{\Gamma h}^{\alpha }\left(s-\tau \right))d\tau =\frac{1}{k_{\Gamma h}^{\alpha }}\;.$$ (30)

Hence, from Eq. (29), a *mechanobiologically equilibrated G&R process* associates with an equilibrium value of the mechano-stimulus function for collagen and smooth muscle in Eq. (10), namely,

$${ϒ}_{\Gamma h}^{\alpha }=1,\;\alpha =m,c.$$ (31)

which, by virtue of Eq. (11), requires either Δ*σ_h_* = 0 = Δ*τ_wh_* or, more generally

$$K_{\Gamma \sigma }^{\alpha }\Delta \sigma_{h}-K_{\Gamma \tau }^{\alpha }\Delta \tau_{wh}=0\;.$$ (32)

As in Ref. 13, we take *τ_w_* = 4*μQ*/(*πa*^3^), with *Q* being the volumetric flow rate and *μ* the blood viscosity, and $\tilde{\sigma }$ in Eq. (9) as the first principal invariant of the mean wall Cauchy stress σ, namely, $\tilde{\sigma }=\text{tr}\;\sigma ≃\sigma_{\theta \theta }+\sigma_{zz}$, where we assume a quasi-plane-stress state for which $\sigma_{rr}/\tilde{\sigma }≃0$. The mean in-plane (biaxial) stresses *σ_θθ_* and *σ_zz_* are given in terms of the distending pressure *P* and the global axial force on the vessel *f_z_*, respectively, through

$$\sigma_{\theta \theta }=\frac{Pa}{h},\;\text{and}\;\sigma_{\text{zz}}=\frac{f_{z}}{\pi h(2a+h)}.$$ (33)

The intramural and shear “over-stresses” expressed in terms of possibly fully evolved (*h*) or original (*o*) homeostatic values (i.e., we allow new homeostatic set-points to evolve) read

$$\Delta \sigma_{h}=\frac{\sigma_{\theta \theta h}+\sigma_{zzh}}{\sigma_{\theta \theta o}+\sigma_{zzo}}-1,\;\text{and}\;\Delta \tau_{wh}=\frac{\tau_{wh}}{\tau_{wo}}-1,$$ (34)

where *a_h_* (present in *σ_θθh_*, *σ_zzh_* and *τ_wh_*), *h_Mh_* and *h_Ah_* (in *σ_θθh_* and *σ_zzh_*), and *f_zh_* (in *σ_zzh_*) are unknowns to be determined for each prescribed alteration in blood pressure, *γ_h_* = *P_h_*/*P_o_*, blood flow, *ε_h_* = *Q_h_*/*Q_o_*, and axial stretch *λ_zh_* = *l_h_*/*l_o_* (note that, in typical biaxial experiments, one usually prescribes axial stretch rather than axial load and it is not possible to infer *f_z_ in vivo*^17^). For simplicity, let smooth muscle and collagen share the same ratio of gain parameters $\eta_{K}=K_{\Gamma \sigma }^{m}/K_{\Gamma \tau }^{m}=K_{\Gamma \sigma }^{c}/K_{\Gamma \tau }^{c}$, despite generally different values of $K_{\Gamma \sigma }^{\alpha }$ and $K_{\Gamma \tau }^{\alpha }$. This means that the perturbation functions ${ϒ}_{\Gamma }^{\alpha }\left(\tau \right)-1$ are proportional,^13^ hence the linearly dependent equations resulting from Eq. (32) reduce to

$$\eta_{K}\left(\frac{\sigma_{\theta \theta h}+\sigma_{zzh}}{\sigma_{\theta \theta o}+\sigma_{zzo}}-1\right)-\left(\frac{\tau_{wh}}{\tau_{wo}}-1\right)=0\;.$$ (35)

So far, we have a single equation with four unknowns, namely Eq. (35) in terms of *a_h_*, *h_Mh_*, *h_Ah_*, and *f_zh_*. The layer-specific Jacobians *J_Mh_* and *J_Ah_*, expressed in terms of the “unchanging” homeostatic stretches in ${\left[F_{\Gamma h}\right]}_{r\theta z}=\text{diag}\;\left[\lambda_{\Gamma rh},\lambda_{\Gamma \theta h},\lambda_{\Gamma zh}\right]$, introduce two additional unknowns (i.e., *J_Mh_* and *J_Ah_*), namely,

$$J_{Mh}=\lambda_{Mrh}\lambda_{M\theta h}\lambda_{zh},\;\text{and}\;J_{Ah}=\lambda_{Arh}\lambda_{A\theta h}\lambda_{zh}\;,$$ (36)

where the radial and circumferential stretches are expressed in terms of *a_h_*, *h_Mh_* and *h_Ah_* through Eqs. (3) and (4), and we assume a common prescribed axial stretch *λ_zh_*. Since the mass of elastin does not change in physiologic adaptations, its layer-specific spatial mass density $\rho_{\Gamma h}^{e}$ becomes directly related to its original spatial mass density $\rho_{\Gamma R}^{e}\equiv \rho_{\Gamma o}^{e}$ through the corresponding volume ratios

$$\rho_{Mh}^{e}=\frac{\rho_{Mo}^{e}}{J_{Mh}},\;\text{and}\;\rho_{Ah}^{e}=\frac{\rho_{Ao}^{e}}{J_{Ah}}\;,$$ (37)

which provide two additional equations but also two additional unknowns, namely $\rho_{Mh}^{e}$ and $\rho_{Ah}^{e}$. Equation (16) particularized to both the media and adventitia yields two more equations, but introduces three more unknowns (namely $\rho_{Mh}^{m},\;\rho_{Mh}^{c}$, and $\rho_{Ah}^{c}$)

$$\rho_{Mh}^{e}+\rho_{Mh}^{m}+\rho_{Mh}^{c}=\rho ,\;\text{and}\;\rho_{Ah}^{e}+\rho_{Ah}^{c}=\rho ,$$ (38)

which leaves four more unknowns than equations. However, as shown previously,^13^ additional relations in terms of the different (evolved homeostatic) spatial mass densities of collagen and smooth muscle can be written as

$$\frac{J_{Mh}\rho_{Mh}^{m}}{\rho_{Mo}^{m}}={\left(\frac{J_{Mh}\rho_{Mh}^{c}}{\rho_{Mo}^{c}}\right)}^{\eta_{q}^{m}\eta_{\Upsilon }^{m}},$$ (39)

and

$$\frac{J_{Ah}\rho_{Ah}^{c}}{\rho_{Ao}^{c}}={\left(\frac{J_{Mh}\rho_{Mh}^{c}}{\rho_{Mo}^{c}}\right)}^{\eta_{q}^{c}\eta_{\Upsilon }^{c}},$$ (40)

where $\eta_{q}^{m}=k_{Mo}^{m}/k_{Mo}^{c},\;\eta_{\Upsilon }^{m}=K_{M\sigma }^{m}/K_{M\sigma }^{c}=K_{M\tau }^{m}/K_{M\tau }^{c},\;\eta_{q}^{c}=k_{Ao}^{c}/k_{Mo}^{c}$, and $\eta_{\Upsilon }^{c}=K_{A\sigma }^{c}/K_{M\sigma }^{c}=K_{A\tau }^{c}/K_{M\tau }^{c}$. We also have, for different layer-specific cohorts *i* of collagen (e.g., circumferential, axial, and symmetric diagonal)^13^

$$\frac{\rho_{\Gamma h}^{c_{i}}}{\rho_{\Gamma o}^{c_{i}}}=\frac{\rho_{\Gamma h}^{c}}{\rho_{\Gamma o}^{c}}\;,$$ (41)

where $\rho_{\Gamma }^{c}=\sum \rho_{\Gamma }^{c_{i}}$. Finally, the system of equations is closed by the global equilibrium equations *σ_θθh_h_h_* = *P_h_a_h_* and *σ_zzh_πh_h_*(2*a_h_* + *h_h_*) = *f_zh_*, where the internal circumferential force *σ_θθh_h_h_* (per unit axial length) in terms of layer-specific stresses in Eqs. (17) and (18), yields

$$\sigma_{M\theta \theta h}h_{Mh}+\sigma_{A\theta \theta h}h_{Ah}=P_{h}a_{h}\;,$$ (42)

and the internal axial force yields, similarly,

$$\sigma_{\text{Mzzh}}\pi h_{Mh}(2a_{h}+h_{Mh})+\sigma_{\text{Azzh}}\pi h_{Ah}(2a_{h}+2h_{Mh}+h_{Ah})=f_{zh}\;.$$ (43)

The different expressions of the mechanobiologically equilibrated, layer-specific stresses to be used in Eqs. (17) and (18) are—see details in Ref. 13

$$\sigma_{\Gamma h}^{\alpha }=\phi_{\Gamma h}^{\alpha }{\hat{\sigma }}^{\alpha }=\frac{\rho_{\Gamma h}^{\alpha }}{\rho }G^{\alpha }{\hat{S}}^{\alpha }(G^{\alpha })G^{\alpha },\;\alpha =m,c,$$ (44)

$$\sigma_{\Gamma h}^{e}=\phi_{\Gamma h}^{e}{\hat{\sigma }}_{\Gamma h}^{e}=\frac{\rho_{\Gamma h}^{e}}{\rho }F_{\Gamma h}G^{e}{\hat{S}}^{e}(C_{\Gamma h}^{e})G^{e}F_{\Gamma h}^{T}\;,$$ (45)

and

$$\sigma_{h}^{act}=\phi_{Mh}^{m}{\hat{\sigma }}_{h}^{act}=\frac{\rho_{Mh}^{m}}{\rho }T_{\max}\left(1-e^{-C^{2}(a_{h})}\right)\left[1-{\left(\frac{\lambda_{M}-1}{\lambda_{M}-\lambda_{0}}\right)}^{2}\right]e_{\theta }⊗e_{\theta }.$$ (46)

Importantly, the nonlinear equations derived in this section do not depend on G&R time *s*, which means that they yield a mechano-adapted solution of the artery for a given sustained altered pressure *P_h_*, flow rate *Q_h_*, and axial stretch *λ_zh_*—hence constituting a truly time- and rate-independent G&R formulation for an idealized bilayered artery. In practice, this system of equations and unknowns may be reduced to five equations and unknowns as explained in Sec. IV E and illustrated in examples addressed above in Results (Sec. II). Of course, other resolution procedures are possible.

### C. The quasi-equilibrium hypothesis

To arrive at the equilibrium condition of Eq. (31), from Eq. (28), we assumed that the artery preserves a static state for a sufficiently long time such that integration of Eq. (29) is exact. The same holds for the equilibrium stresses of cohorts of smooth muscle and collagen given in Eq. (44), which are obtained upon integration of the corresponding integral-type Eqs. (19) and (21). Considering Eq. (29), we see that $k_{\Gamma }^{\alpha }$ and ${ϒ}_{\Gamma }^{\alpha }$, as given in Eqs. (8) and (11), become constant if the wall stress metric $\tilde{\sigma }\equiv \sigma_{\theta \theta }+\sigma_{zz}$ and the shear stress *τ_w_* remain constant over long times. Taking into account Eqs. (33) and (34), this requires that the applied external loads *P*, *Q*, and *λ_z_* remain constant over long times, which are, indeed, the ultimate variables that stimulate the G&R response in the present mechanoadaptive case.

In what follows, we relax this equilibrium hypothesis to introduce the definition of a quasi-equilibrium state, which we illustrate by comparing three cases: *I*, *II*, and *III*. Assume a survival function $q_{0}^{\alpha }\left(\tau \right)=q_{\Gamma }^{\alpha }\left(s_{0},\tau \right)$, as given in Eq. (7), as a function of the deposition time *τ* ≤ *s*_0_ for a fixed G&R time *s*_0_. Let $T^{\alpha }:=[s_{0}-\Delta s^{\alpha },s_{0}]$ be a proper integration domain such that $q_{0}^{\alpha }\in [0^{+},1]$, with 0^+^ a sufficiently small value such that longer integrations of $q_{0}^{\alpha }\left(\tau \right)$ beyond *s*_0_–Δ*s^α^* yield (additional) negligible contributions. Consider, first, the evolution over time of a generic nondimensional external insult $\xi_{ext}^{I}\left(\tau \right)$ that, qualitatively, includes any mechanical alteration such as $P\left(\tau \right)/P\left(s_{0}\right)\neq 1,\;Q\left(\tau \right)/Q\left(s_{0}\right)\neq 1$, or $\lambda_{z}\left(\tau \right)/\lambda_{z}\left(s_{0}\right)\neq 1$. Obviously, if the rate of change of $q_{0}^{\alpha }\left(\tau \right)$ and $\xi_{ext}^{I}\left(\tau \right)$ are comparable within $T^{\alpha }$, namely, ${\dot{q}}_{0}^{\alpha }=dq_{0}^{\alpha }/d\tau ∼d\xi_{ext}^{I}/d\tau ={\dot{\xi }}_{ext}^{I}$ for $\tau \in T^{\alpha }$, then the associated variations of $k_{\Gamma }^{\alpha }\left(\tau \right)$ and ${ϒ}_{\Gamma }^{\alpha }\left(\tau \right)$ over time [assuming gain parameters $K_{\Gamma \sigma }^{\alpha }$ and $K_{\Gamma \tau }^{\alpha }$ of order unity, see Eq. (11)] cannot be neglected in the integral of Eq. (28), and the general formulation cannot be simplified. In this case (*I*), one needs to keep track the history of all the involved variables, at least within $T^{\alpha }$, to compute the G&R solution at any time *s*_0_—that is, one must employ the full (hereditary integral) constrained mixture formulation.^2,18^

Consider here, however, a case (*II*) for which ${\dot{\xi }}_{ext}^{II}=0$ within $T^{\alpha }$. Then, if the G&R response is mechanobiologically stable^20^ and has relaxed fully, then the variables $k_{\Gamma }^{\alpha }$ and ${ϒ}_{\Gamma }^{\alpha }$ reach, after the corresponding characteristic G&R period, constant values $k_{\Gamma h}^{\alpha }$ and ${ϒ}_{\Gamma h}^{\alpha }=1$ within the relevant integration domain $T^{\alpha }$ as well. The artery thus reaches a (new) tissue maintenance state, for which $\rho_{\Gamma R}^{\alpha }\to \rho_{\Gamma Rh}^{\alpha }$ is also constant, leading to the result of Eq. (29) and, eventually, to the mechanobiologically equilibrated formulation detailed above. As explained in,^13^ a characteristic time scale for both mass removal and production processes is given by $s_{G\&R}=1/\min\{k_{\Gamma o}^{\alpha },k^{act}\}$, hence mechanobiological equilibrium is attained at *s *≫* s_G&R_*. Recall that gain parameters $K_{\Gamma \sigma }^{\alpha }≁1$ and $K_{\Gamma \tau }^{\alpha }≁1$ can modify the value of *s_G&R_* quantitatively.

Another important case (*III*) is one for which the rate of change of the external stimuli ${\dot{\xi }}_{ext}^{III}$ is much less than the rate of change of the removal function ${\dot{q}}_{0}^{\alpha }$ within $T^{\alpha }$. From a mathematical standpoint, the variables of any integrands can then be regarded constant within the integration domain $T^{\alpha }$, hence we recover the previous equilibrium formulation approximately at each G&R time *s*, namely a quasi-equilibrium formulation valid during the evolution. For example, the integral of Eq. (28) yields, for slow ${\dot{\xi }}_{ext}$, at any G&R time *s* [cf. Eq. (29)]

$$\rho_{\Gamma R}^{\alpha }\left(s\right)≃k_{\Gamma }^{\alpha }\left(s\right)\rho_{\Gamma R}^{\alpha }\left(s\right){ϒ}_{\Gamma }^{\alpha }\left(s\right)\int_{s-\Delta s^{\alpha }}^{s}q_{\Gamma }^{\alpha }\left(s,\tau \right)d\tau ≃\rho_{\Gamma R}^{\alpha }\left(s\right){ϒ}_{\Gamma }^{\alpha }\left(s\right),\;\forall s,$$ (47)

whereby the stimulus function resolves, that is, [cf. Eq. (31)]

$${ϒ}_{\Gamma }^{\alpha }\left(s\right)≃1,\;\forall s\;.$$ (48)

From a physical standpoint, we can equivalently focus on the evolution of an arbitrary mass deposited at a fixed time *τ*_0_ that is removed gradually during ${\bar{T}}^{\alpha }:=[\tau_{0},\tau_{0}+\Delta s^{\alpha }]$. Hence, if quasi-equilibrium conditions are satisfied, the differential mass senses an almost constant mechanical environment during its timespan Δ*s^α^*, undergoing then a so-called quasi-steady evolution. The evolution is said to be mechanobiologically quasi-equilibrated (rather than mechanobiologically equilibrated) because the external stimuli $\xi_{ext}^{III}$ may be different at different G&R times over a time scale much longer than Δ*s^α^*; that is, $\xi_{ext}^{III}\left(s_{0}\right)≃\xi_{ext}^{III}\left(s_{1}\right)$ for $s_{1}-s_{0}∼\Delta s^{\alpha }$, but $\xi_{ext}^{III}\left(s_{0}\right)≄\xi_{ext}^{III}\left(s_{1}\right)$ for *s*_1_ – *s*_0_ ≫ Δ*s^α^*, in general.

In summary, if ${\dot{\xi }}_{ext}\ll {\dot{q}}_{0}^{\alpha }$ within $T^{\alpha }$, the time-independent formulation derived in Sec. IV B remains valid throughout the mechanoadaptation, with G&R time *s* playing the role of a parameter while the external stimuli is able to change over a much longer period. Using the decay function of Eq. (7), we obtain (by the Leibniz integral rule)

$$\frac{dq_{0}^{\alpha }\left(\tau \right)}{d\tau }=\text{exp}\;\left(-\int_{\tau }^{s_{0}}k_{\Gamma }^{\alpha }\left(t\right)dt\right)\frac{d}{d\tau }\left(-\int_{\tau }^{s_{0}}k_{\Gamma }^{\alpha }\left(t\right)dt\right)=q_{0}^{\alpha }\left(\tau \right)k^{\alpha }\left(\tau \right),$$ (49)

so a characteristic mass-specific rate of removal at a given G&R time *s* is ${\dot{q}}_{0}^{\alpha }\left(s\right)=k_{\Gamma }^{\alpha }\left(s\right)∼k_{\Gamma o}^{\alpha }$. If we include the evolution of the active reference length for the active stress contribution of smooth muscle, the quasi-equilibrium formulation is valid then if ${\dot{\xi }}_{ext}\left(s\right)\equiv k_{ext}\left(s\right)\ll k_{G\&R}=\min\{k_{\Gamma o}^{\alpha },k^{act}\},\;\forall s$. Note that this is equivalent to saying that a characteristic time for G&R, namely *s_G&R_* = 1/*k_G&R_*, is much shorter than a characteristic time of change of the (normalized) external loads, namely *s_ext_* = 1/*k_ext_*.

### D. Temporal nondimensionalization of the full model

The concept of a characteristic time scale for a G&R response of a living soft tissue encourages us to nondimensionalize in the time domain in the full constrained mixture model outlined in Sec. IV A. Given the characteristic time $s_{G\&R}=1/\min\{k_{\Gamma o}^{\alpha },k^{act}\}$, we first define dimensionless rate parameters

$${\tilde{k}}_{\Gamma o}^{\alpha }=k_{\Gamma o}^{\alpha }\cdot s_{G\&R},\;\alpha =m,c,$$ (50)

and

$${\tilde{k}}^{act}=k^{act}\cdot s_{G\&R},$$ (51)

as well as dimensionless time variables

$$\tilde{s}=s/s_{G\&R},\;\tilde{\tau }=\tau /s_{G\&R},\;\tilde{t}=t/s_{G\&R},$$ (52)

such that we have equivalent products between original and dimensionless rate parameters and time variables of the type

$$k_{\Gamma o}^{\alpha }\cdot s=(k_{\Gamma o}^{\alpha }\cdot s_{G\&R})\cdot (s/s_{G\&R})={\tilde{k}}_{\Gamma o}^{\alpha }\cdot \tilde{s},$$ (53)

and so forth. We can then obtain equivalent integrals for Eq. (7)

$$q_{\Gamma }^{\alpha }\left(s,\tau \right)=\text{exp}\;\left(-\int_{\tau }^{s}k_{\Gamma }^{\alpha }\left(t\right)dt\right)=\text{exp}\;\left(-\int_{\tilde{\tau }}^{\tilde{s}}{\tilde{k}}_{\Gamma }^{\alpha }\left(\tilde{t}\right)d\tilde{t}\right)=q_{\Gamma }^{\alpha }\left(\tilde{s},\tilde{\tau }\right),$$ (54)

where [cf. Eq. (8)]

$${\tilde{k}}_{\Gamma }^{\alpha }=k_{\Gamma }^{\alpha }\cdot s_{G\&R},$$ (55)

and for Eq. (12)

$$\rho_{\Gamma R}^{\alpha }\left(s\right)=\int_{-\infty }^{s}m_{\Gamma R}^{\alpha }\left(\tau \right)q_{\Gamma }^{\alpha }\left(s,\tau \right)d\tau =\int_{-\infty }^{\tilde{s}}{\tilde{m}}_{\Gamma R}^{\alpha }\left(\tilde{\tau }\right)q_{\Gamma }^{\alpha }\left(\tilde{s},\tilde{\tau }\right)d\tilde{\tau }=\rho_{\Gamma R}^{\alpha }\left(\tilde{s}\right),$$ (56)

where [cf. Eq. (10)]

$${\tilde{m}}_{\Gamma R}^{\alpha }=m_{\Gamma R}^{\alpha }\cdot s_{G\&R},$$ (57)

and for Eq. (13)

$$W_{\Gamma R}^{\alpha }(s)=\frac{1}{\rho }\int_{-\infty }^{s}m_{\Gamma R}^{\alpha }\left(\tau \right)q_{\Gamma }^{\alpha }\left(s,\tau \right){\hat{W}}^{\alpha }(C_{\Gamma n\left(\tau \right)}^{\alpha }(s))d\tau ,$$ (58)

$$=\frac{1}{\rho }\int_{-\infty }^{\tilde{s}}{\tilde{m}}_{\Gamma R}^{\alpha }\left(\tilde{\tau }\right)q_{\Gamma }^{\alpha }\left(\tilde{s},\tilde{\tau }\right){\hat{W}}^{\alpha }(C_{\Gamma n\left(\tilde{\tau }\right)}^{\alpha }(\tilde{s}))d\tilde{\tau }=W_{\Gamma R}^{\alpha }(\tilde{s}).$$ (59)

Finally, the time-dimensionless counterpart of the evolution equation in the rate form of Eq. (26) reads

$$\frac{1}{k^{act}}\frac{da^{act}\left(s\right)}{ds}=a\left(s\right)-a^{act}\left(s\right)=a\left(\tilde{s}\right)-a^{act}\left(\tilde{s}\right)=\frac{da^{act}\left(\tilde{s}\right)}{d\tilde{s}}\frac{1}{{\tilde{k}}^{act}}\;.$$ (60)

Thus, every time-dependent evolution equation can be equivalently expressed in terms of dimensionless rate parameters and time variables. This means that, once a time-dependent G&R response is computed for a given tissue, say tissue *B*, with characteristic G&R time $s_{G\&R}^{B}$, rate-type parameters *k^actB^* and $k_{\Gamma o}^{\alpha B},\;\alpha =m,c$, and prescribed external insults $\xi_{ext}^{B}\left(s^{B}\right)$, the solution represented in terms of the dimensionless time $\tilde{s}=s^{B}/s_{G\&R}^{B}$ is common for any other tissue, say *D*, with proportional rate-type parameters $k^{\text{actD}}=\beta k^{\text{actB}}$ and $k_{\Gamma o}^{\alpha D}=\beta k_{\Gamma o}^{\alpha B}$, with $\beta =s_{G\&R}^{B}/s_{G\&R}^{D}$, and prescribed external insults $\xi_{ext}^{D}\left(s^{D}\right)=\xi_{ext}^{B}\left(s^{B}/\beta \right)$, with common remaining material properties, because they share the same time-dimensionless formulation.

### E. Resolution procedure

For the pre-integrated model of Sec. IV B, we will solve the system of nonlinear equations formed by Eqs. (35), (38)_1_, (38)_2_, (42), and (43), where the unknowns are the (potentially new) homeostatic inner radius *a_h_*, layer thicknesses *h_Mh_* and *h_Ah_*, spatial mass density of collagen within the media $\rho_{Mh}^{c}$, and global axial force *f_zh_*, with other variables expressed easily in terms of the selected unknowns. The resulting system of equations is time-independent and so too its outcome.

The hyperelastic mechanical response of elastin is modelled using a neoHookean relation

$${\hat{W}}^{e}(C^{e}(s))=\frac{c^{e}}{2}\left(C^{e}(s):I-3\right)\;,$$ (61)

with *c^e^* being the shear modulus. Hyperelastic responses of both smooth muscle and collagen are modelled using Fung-type relations

$${\hat{W}}^{\alpha }(\lambda_{n\left(\tau \right)}^{\alpha }(s))=\frac{c_{1}^{\alpha }}{4c_{2}^{\alpha }}\left[e^{c_{2}^{\alpha }{\left(\lambda_{n\left(\tau \right)}^{\alpha 2}(s)-1\right)}^{2}}-1\right],\;\alpha =m,c\;,$$ (62)

where $c_{1}^{\alpha }$ (dimensions of stress) and $c_{2}^{\alpha }$ (dimensionless) are material parameters, and $\lambda_{n\left(\tau \right)}^{\alpha }(s)$ is the corresponding fiber stretch. We consider four collagen fiber families in both the media and adventitia: one oriented circumferentially (labelled with *θ*), one oriented axially (*z*), and two oriented in symmetric diagonal (*d*) directions  ± *α*_0_ with respect to the axial direction. The contributions of circumferential collagen and smooth muscle are combined in the media^16^ (referred to as medial circumferential smooth muscle *m*). Medial and adventitial collagen are assumed to share the same hyperelastic, rate, and gain constants, the difference in contributions coming from different mass fractions. All the material parameters needed to obtain both time-dependent and time-independent solutions are listed in Table I. The specific values of the parameters are best-fit values determined from *in vitro* biaxial data from passive elastic arteries of mice.^21^ In order to show the full consistency between both formulations when they include all possible contributions to stress, however, we take additional values for the active response of smooth muscle (Table I). Finally, no ethics approval was required since all work was numerical.
